# Relationships between subjective experience, electroencephalogram, and heart rate variability during a series of cosmetic behavior

**DOI:** 10.3389/fpsyg.2024.1225737

**Published:** 2024-05-14

**Authors:** Hiroki Moriya, Akiko Machida, Taro Munakata, Tomomitsu Herai, Keiko Tagai

**Affiliations:** ^1^Centan Inc., Minato-ku, Japan; ^2^MIRAI Technology Institute, Shiseido Co., Ltd., Yokohama, Japan

**Keywords:** skincare, makeup, cosmetic products, emotion, electroencephalogram, heart rate variability

## Abstract

**Introduction:**

Cosmetic behavior is an important daily activity, especially for women, because it increases visual attractiveness, self-confidence, and positive emotions. However, it is unknown whether a relationship exists between physiological measures and subjective experiences during the series of cosmetic behaviors.

**Methods:**

Electroencephalograms (EEG) and electrocardiograms (ECG) from thirty female participants who were asked to look in a mirror after applying skincare, as well as base, eye, cheek, and lip makeup were recorded. The price range of cosmetic products was also considered. Subjective evaluations of the skin surface, emotions, and self-confidence were equally measured after looking in the mirror at each step of the cosmetic behavior. Linear mixed models were fitted to examine whether the subjective experience could be explained by the variety of cosmetic products and/or physiological responses.

**Results:**

The subjective evaluation was summarized into the following three factors using a factor analysis: self-confidence, hedonic perception, and negative emotion. Each theta-band (4–6 Hz) power, alpha-band (8–13 Hz) power of the EEG, and heart rate variability measures were subjected to a principal component analysis separately. The linear mixed models indicated that the variation in the self-confidence score and the negative emotion score was explained only by the steps of cosmetic behaviors, that is, self-confidence increased while negative emotions decreased as the steps of cosmetic behaviors proceeded. On the other hand, the hedonic perception score was explained by the interaction of the steps of cosmetic behaviors and price, indicating that positive tactile perception and positive emotion were higher when luxury cosmetic products were applied than when affordable products were applied. Furthermore, the model indicated that the hedonic perception score was positively associated with the alpha-band power over occipital sites whereas sympathetic nervous system activity was negatively associated with the alpha-band power over lateral central sites.

**Discussion:**

These results suggest that positive perceptual and emotional experiences are associated with greater attention to somatosensory information than to visual information and sympathetic autonomic nervous system activities. The current results also emphasize the possibility of using physiological measurements as objective measures of cosmetic behavior.

## 1 Introduction

In today’s marketplace, a wide variety of cosmetic products with different textures, colors, functional ingredients, and brands are available. Cosmetic behavior is a daily activity for cosmetic users and is an important part of their personal and social daily lives. Although applying cosmetics is a habit that takes only a few minutes of the users’ time, it not only improves their skin and health but also affects their mindset. Previous studies have revealed that one of the socio-psychological functions of makeup is to improve the social evaluation and/or perception of others toward the user by making the user’s appearance more physically attractive (Etcoff et al., 2011).

The effect of cosmetics on physical attractiveness is an example of the traditional social role of cosmetic behavior, especially when seeking a job or spouse, or participating in ceremonies. This idea is supported by a growing body of evidence, which has revealed that facial makeup can improve the attractiveness evaluation of an individual’s face by observers (Nash et al., 2006), and can facilitate prosocial activity from other people (Jacob et al., 2010). Several psychophysiological studies suggest that the positive influence of makeup might be due to the modulated perceptual process of an individual’s face, which involves impression formation by applying cosmetics (Tagai et al., 2016, 2017). These studies indicate that facial makeup affects perceptual, cognitive, and emotional processes that may involve social interactions with others.

The effect of makeup is not only a positive evaluation of the impressions of others, such as an increase in attractiveness but also the psychological satisfaction gained from seeing oneself with facial makeup. It has been reported that everyday makeup not only increases facial-appearance satisfaction but also increases one’s sense of attractiveness (Cash et al., 1989). A previous study revealed that more than half of the women responded that makeup had a positive impact on their self-esteem and confidence (Kosmala et al., 2019). Regarding neurophysiological effects, research has revealed that the amplitudes of several event-related potential components (i.e., N170, early posterior negativity, and late positive potential), which are thought to reflect visual attention, increase when viewing faces with makeup rather than without makeup (Arai and Nittono, 2022). It was interpreted that this rewarding value is added not only by others’ makeup but also by watching themselves use makeup.

Other aspects of cosmetic behavior, as well as changes in appearance, are thought to change consumers’ experiences and the associated physiological activities. Previous studies have shown that using skincare products can reduce psychological stress and induce relaxed and comfortable psychological states, which are measured subjectively as well as physiologically such as through the responses of the stress hormone cortisol and the immune substance S-IgA (Hirao, 2002), electroencephalogram (EEG) (Bouhout et al., 2023), and cerebral blood flow, which is measured by functional near-infrared spectroscopy (fNIRS) (Tanida et al., 2017). Recently, several studies have demonstrated that the activities of brain regions in response to self-touching are possible measures of cosmetic behaviors such as consumers’ knowledge and attachment to a cosmetic product (Hirao et al., 2020, 2021; Kikuchi et al., 2021). In terms of attachment, Kikuchi et al. showed that the activities of the dorsal raphe nucleus and periaqueductal gray matter were higher when participants used serum with attachment than when they used serum without attachment. In terms of knowledge of cosmetic products, Hirao et al. showed that the coupling between the ventral striatum, dorsomedial prefrontal cortex, somatosensory area (Hirao et al., 2021), prefrontal cortex, and ventral striatum (Hirao et al., 2020) while hand rubbing skincare creams increased when a luxury cue was given. On the other hand, relationships between physiological activities and subjective evaluation when using cosmetic products have also been examined (Kawabata Duncan et al., 2019; Gabriel et al., 2021). Gabriel et al. (2021) examined the effect of the viscosity of cosmetic products applied to participants’ hands on their emotional response using EEG features. They found that a low viscous product was preferred to a high viscous product. Furthermore, they found that right-biased asymmetric frontal alpha (8–12 Hz) EEG activity, thought to be a neural index of approach motivation (e.g., Harmon-Jones and Gable, 2018), was greater in the low-viscosity cream than in the high-viscosity cream. Using fNIRS, Tanida et al. (2017) reported that left-dominant activity in the cerebral blood flow responses in the prefrontal cortex was observed during the application of a smooth lipstick. Kawabata Duncan et al. (2019) showed that activation of the right dorsolateral prefrontal cortex during the application of a foundation was positively correlated with the willingness-to-pay score for the given foundation in participants who frequently used the foundation (six or more days in a week). An intra-subject correlation between the right dorsolateral prefrontal cortex (dlPFC), which is regarded as possessing the function of making value judgments by referring to experiences, and the willingness to pay high prices was found during a single real-life use of lipstick that changed the quality level and color of preference (Hirabayashi et al., 2021). In facial skincare, there is a decrease in the ratio of low-frequency and high-frequency powers (LF/HF ratio) of heart rate variability (HRV), indicating cardiac relaxation, and an increase in the alpha/beta ratio, indicating brain relaxation (Bouhout et al., 2023).

The aforementioned evidence shows that cosmetic behavior can influence consumers’ experiences, such as tactile perception of the skin surface, visual perception of the face, and emotions. Furthermore, psychophysiological evidence suggests that activities in wide cortical/subcortical areas that support sensory, rewarding, and emotional processing are involved in the construction of experiences that accompany cosmetic behavior. This evidence strongly suggests that physiological responses can be an objective measure of user experience during cosmetic behaviors. However, although cosmetic behavior consists of a series of cosmetic products, such as skincare, foundation, and eye makeup, most previous studies have examined the psychophysiological effects of a specific cosmetic product, and whether previous findings can be generalized to cosmetic behavior at large remains unclear.

Emotional responses involve changes in subjective experiences and physiological activities. Previous neuroscientific and psychophysiological studies have shown that the central and autonomic nervous systems are the primary neural bases involved in the generation and processing of emotions (Kreibig, 2010; Kragel and Labar, 2014, 2016). Recent studies have also suggested that emotions can be predicted by the central nervous system activity features obtained from EEG features (e.g., Çelikkanat et al., 2017) and autonomic nervous system activity, including heart-rate features obtained from electrocardiograms (ECG) (e.g., Kragel and Labar, 2013). More recent studies indicated that emotions elicited by a standardized emotion elicitation stimulus can be accurately classified by using non-linear EEG features (e.g., Zangeneh Soroush et al., 2018a,b, 2020).

Perceptions of tactile and/or visual input while applying cosmetics are also important aspects of subjective experiences during cosmetic behavior. For example, the compounds that are included in a cosmetic product can change several physical properties of the product such as smoothness and color, and can thus alter users’ tactile and visual perceptual experiences. Such kind of perceptual experiences may be accompanied by a feeling of goodness and/or badness of the products, depending on the context of the cosmetic behavior, such as users’ purpose of using the cosmetics (e.g., for daily work or for attending a party), preference, and prior knowledge about the products. However, little is known about the perceptual properties of the subjective experience and their physiological correlation during a series of cosmetic behaviors. EEG is also thought to reflect perceptual processes. In the case of visual processing, alpha band power over occipital electrode sites is shown to decrease when a visual input occurs (Fries et al., 2008; Bollimunta et al., 2011). Similar to the visual modality, alpha band power over lateral central and parietal electrode sites is shown to decrease in response to the occurrence of tactile inputs (Cheyne et al., 2003; Singh et al., 2014). This alpha suppression in response to external stimuli is modulated by emotion. Previous studies have demonstrated that alpha power suppression which occurs after picture onset increased (i.e., less alpha power) in response to a strong negative emotional picture (e.g., mutilated body) or a strong positive emotional picture (e.g., romantic scene) was presented as compared to a neutral emotional picture (for a review, see Codispoti et al., 2023). These effects of emotional intensity on the brain activities related to perception are interpreted as increased attentional resource allocation to the environmental stimulus that contains emotional information (e.g., Bradley et al., 2003; Schupp et al., 2004). The heart rate variabilities are also shown to reflect perception and emotion which involve a greater deceleration of the heart rate in response to an unpleasant stimulus as compared to a neutral or a pleasant stimulus, as indicated in several previous studies (Lang et al., 1993; Bradley and Lang, 2000; Cuthbert et al., 2000). Furthermore, Lang et al. (1993) indicated that acceleration of the heart rate positively correlated with participants’ pleasantness judgment. Given that the activation of the sympathetic nervous system and parasympathetic nervous system accelerates and decelerates the heart rate, these previous findings suggest that the activation of the sympathetic nervous system or the inhibition of the parasympathetic nervous system activity is related to the construction of positive emotion.

We aim to apply these findings to the development of cosmetics to provide greater emotional value to consumers in their daily lives. However, cosmetic behavior involves a series of steps starting from face washing, skincare, and makeup, and few studies have examined the transition of emotional and physiological changes in a series of daily steps of cosmetic behavior. In addition, the choice of cosmetics depends on the consumer’s purpose of use, preferences, and economic status, involving a wide range of brands and price variations, ranging from affordable to expensive. Based on previous findings, it can be inferred that the emotional value gained from using cosmetics has something in common with its usage regardless of the brand and price, but luxury brands have a higher added value to consumers. In the present study, we recorded subjective and physiological measures of female participants who used a series of cosmetic products with different price ranges to examine the effects of price, cosmetic steps, and participants’ physiological responses on their subjective emotional responses. Given that the order of application of cosmetics in realistic circumstances is almost fixed and it is difficult to use an ideal experimental design in which the order of the condition should be fully randomized, we used a quasi-experimental design in which the participants apply a series of cosmetics in a fixed order to replicate a naturalistic and realistic cosmetic behavior. In terms of the definition of emotional values, to simplify experimental design, we only focused to object values of the cosmetic items; specifically, we manipulated the objective values of the cosmetic items by comparing participants’ subjective experiences among different price range, i.e., affordable and luxury brands, in which objective value is high and low, respectively. We examined what kind of user experience emerged during cosmetic behavior by using a questionnaire including items that are related to emotional and perceptual experience. Furthermore, we explored whether both cosmetic product categories differed between price and steps and/or whether the physiological response explained the variation in subjective experience by comparing two statistical models: one including only the cosmetic categories as independent variables, and the other including both cosmetic product categories and physiological responses as independent variables.

Unlike statistical models of emotion and emotion recognition studies that have been validated using the standardized experimental procedure and the standardized stimulus dataset, the purpose of the current study is to examine the relationships between subjective experience and psychophysiological measurements recorded from participants who were applying a series of cosmetics as a routine procedure. Except for fatigue detection during driving (e.g., Ren et al., 2021), there are still few studies that predict emotions experienced in everyday behavior from physiological indices. We therefore recognize the need to accumulate findings even if the procedures and methods are not yet sophisticated. We believe that such findings can be used to further improve the psychological well-being of consumers based on their daily behavior and can be utilized as evidence of the psychological usefulness of cosmetic actions and in the development of cosmetic products.

Although our purpose is exploratory in nature, we hypothesize that central and autonomic nervous system activities play a role in the emergence of subjective emotional responses. More specifically, user experience during cosmetic behavior can be explained by both cosmetic products and physiological responses. For the effect of cosmetic products, we hypothesized that using luxury brands in which objective value is higher than in affordable brands, can elicits a more positive user experience. For the physiological responses, both EEG responses, which are related to emotion and attention such as alpha suppression, and HRV, which are related to sympathetic nervous system activities, can explain the variations in positive user experiences. We believe that our study makes a significant contribution to the literature because, to the best of our knowledge, this is the first study to investigate and reveal (1) how the variation of cosmetic products affects participants’ perceptions and emotions during the cosmetic behavior, and (2) the relationships between the physiological responses and the subjective experiences during the naturalistic cosmetic behavior.

## 2 Materials and methods

### 2.1 Participants

Thirty healthy female participants aged between 22 to 35 years (median: 29 years) with normal or corrected-to-normal vision participated in the experiment. All participants were right-handed based on a self-report. For economic reasons, the sample size was not based on a power analysis, but instead, we determined the sample size based on previous related studies such as that of Koelstra et al. (2012). Prior to participation, each participant provided written informed consent. The participants were screened and recruited from a participant database registered with a research company as per their health and daily cosmetic use. More specifically, female participants who met the following criteria based on self-reports were included: have a normal or corrected-to-normal vision, have no history of any neurological and mental disorders, have not received dermatological treatment within 1 year, and use skincare, base makeup, eye makeup, lipstick, and cheek makeup products at least 4 days/week. The experimental design and procedure were approved by the ethics committee of the MIRAI Technology Institute, Shiseido Co., Ltd (the human study number is C01967).

### 2.2 Cosmetic materials

Forty cosmetic products from three brands were selected based on their low or high price level (hereafter, affordable and luxury, respectively) for four cosmetic steps (skincare, makeup base and foundation as base makeup, eye makeup, and lips and cheeks makeup). Detailed information of the cosmetic products used in the current study is summarized in Supplementary Table S1. For each cosmetic step, several variants based on color, material type (e.g., liquid or powder type), and function (e.g., whitening or moisturizing) exist. All the samples were manufactured by Shiseido Co., Ltd. For the affordable and luxury conditions, the cosmetic items were selected from low-price and high-price cosmetic items, respectively. The prices of the products used in the affordable and luxury conditions ranged from 700 to 1,500 and 2,000 to 30,000 Japanese yen, respectively. The prices of the products varied in both the affordable and the luxury conditions due to a price practice in the cosmetic manufacturers. For instance, the price of eye makeup products is low while the price of foundation/base makeup products is high.

### 2.3 Procedure

**Figure 1:**
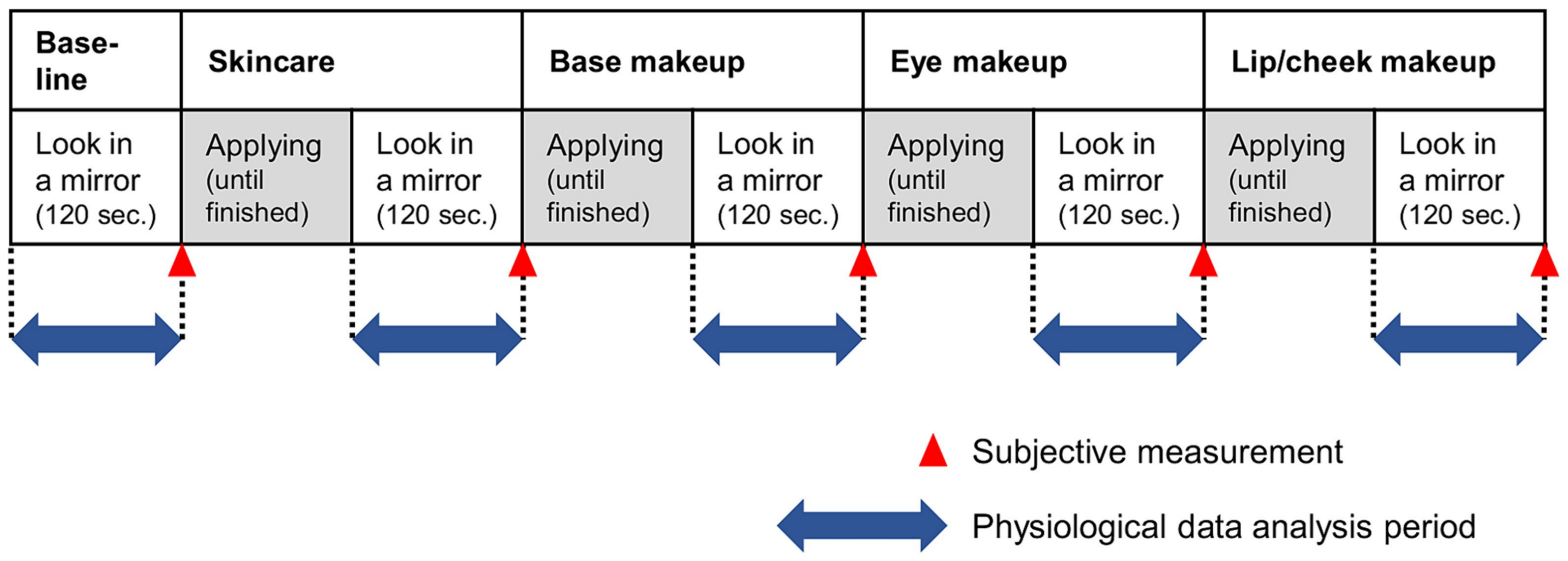
Protocol of the application of cosmetic products used in the current study. Each participant completed the protocol for each price condition.

The experiment was conducted in two rooms at Shiseido Global Innovation Center. Two participants participated simultaneously in each room. They completed two sessions of cosmetic application tasks (reported here), followed by a two-alternative forced-choice task for cosmetic products (not reported here). At the beginning of the experiment, each participants asked to choose of cosmetic items for each price level and cosmetic step, based on their preference of colors and texture. Participants were allowed to test the products’ color and texture by applying the products on their back of the hands. By this procedure, two sets of the series of cosmetic steps (skincare, base makeup, eye makeup, and lip and cheek makeup) were created for each participant, one set consisted of affordable price level, and the other consisted of luxury price level. The procedure for the cosmetic behavior task is shown in Figure 1. The task consisted of two cosmetic application task sessions in which the price of the cosmetic products differed; that is, the cosmetic products of affordable brands were presented in one session (affordable session) and the cosmetic products of a luxury brand were presented in the other section (luxury session). At the beginning of each session, participants were asked to wipe their facial makeup. After removing their makeup, the participants were asked to look at their natural faces for 120 s and then answered questions about the subjective experience. Afterward, the participants applied cosmetic products in the following order: skincare, base, eye, lip, and cheek makeup. At the beginning of each cosmetic step, three to eight cosmetic products were included in the cosmetic product types and prices. The participants were asked to choose one or more cosmetic products from among the given options. After choosing the products, the participants applied cosmetics to their faces. In both the affordable and luxury sessions, the participants were asked to apply cosmetics as if they were prepared to go out. Although there were no time limits to finish each cosmetic step, a short beeping sound at each step was presented 5 min after the participants started applying the cosmetics to notify them of the elapsed time. Participants were allowed to continue applying the cosmetics after this notification. After finishing the cosmetic steps, they were asked to watch their face in a mirror placed on a desk in front of them and to think about the effects of wearing the cosmetics for 120 s. At the end of each cosmetic step, the participants answered questions about their emotions and perceptions using a visual analog scale (see below).

### 2.4 Subjective measurements

To quantify the subjective perception and emotion elicited for cosmetic behavior, three questions related to the use of cosmetic products (the product is easy to use, feels good to touch the skin, and a good impression of the entire face), and ten questions related to perception and emotion (feel cheerful, calm, refreshed, confident, concentrated, satisfied, anxious, unpleasant, want to see someone, and feeling sleepy) were raised. The questionnaire items were based on a questionnaire used in our study (Sato, 2020) and two-dimensional affective state questionnaires (e.g., Russell et al., 1989). The participants were asked to answer every question by choosing an option that represents their feeling or perception via visual analog scales (i.e., clicking on ten-centimeter horizontal lines presented on the monitor, ranging from not at all (left end) to extremely (right end)). The answers to each question were quantified on a 0.1-point resolution, from 0.0 (not at all) to 100.0 (extremely), for each participant, price condition, and cosmetic step.

### 2.5 Physiological data recording and analysis

EEG signals were recorded from 18 electrode sites (F3/4, F7/8, C3/4, T7/8, P3/4, P7/8, O1/2, Fz, Cz, Pz, and Oz) referenced to the left mastoid (M1). Electrooculographic signals were recorded from an electrode placed at AF3. Electrocardiographic signals were recorded from electrodes placed over the manubrium sterni and left ventricle using a bipolar montage. The physiological signals were recorded at a sampling rate of 1 kHz. The electrode impedance was maintained below 20 kΩ. For half of the participants, Polymate AP1000 was used and for the other half of the participants, Polymate V (TEAC Ltd. Tokyo, Japan) was used for signal recording. A preliminary test demonstrated that the signals recorded simultaneously by these amplifiers were extremely high (coherence >0.8). The signals were preprocessed by MNE-Python 0.23 (Gramfort et al., 2013). Offline, a notch filter of 50 Hz and a band-pass filter of 1–30 Hz, were applied to each physiological signal. For each participant and price condition, the EEG signals were re-referenced to the average reference and segmented into five 120 s epochs where participants had been watching their faces before ocular artifact removal using extended-Infomax independent component analysis (ICA). The independent components (ICs) were visually inspected, and the ocular artifact ICs identified based on the power spectrum and topographical maps were removed manually. The vertical ocular movement and/or blink artifact ICs were identified as having a greater power spectrum, density was found to be less than 5 Hz, and a greater topographical activation was found around the bilateral frontal electrode site (especially in F3 and F4). The horizontal ocular movement ICs were identified as a greater power spectrum density of the source activation, and a laterally inverted topographical activation pattern was found over the lateral frontal electrode site (F7 and F8).

The ECG signal was also segmented into five 120-s epochs for each participant and price condition. For each participant, price condition, and cosmetic behavior step, R-peaks were detected in the ECG signal.

The physiological features were theta-and alpha-band power of EEG and mean inter-beat-interval (IBI), coefficient of variation of R-R interval (CVRR), a root-mean-squared standard deviation of R-R interval (RMSSD), a standard deviation of R-R interval (SDNN), low-frequency power (LF, 0.04–0.15 Hz), high-frequency power (HF, 0.15–0.4 Hz), a ratio of low-frequency and high-frequency (LF/HF), cardiovascular vagal index (CVI), and cardiovascular sympathetic index (CSI). For each participant, price condition, cosmetic step, and electrode site, a short-time Fourier transformation (2-s epochs with a 1-s sliding window) was applied to the EEG signals, and the logarithmic (base 10) transformed mean power of the theta (4–7 Hz) and alpha (8–13 Hz) bands. The heart-rate variability features were estimated for each participant, price condition, and cosmetic step using the HRV analysis Python package.

### 2.6 Statistical analysis

**Figure 2:**
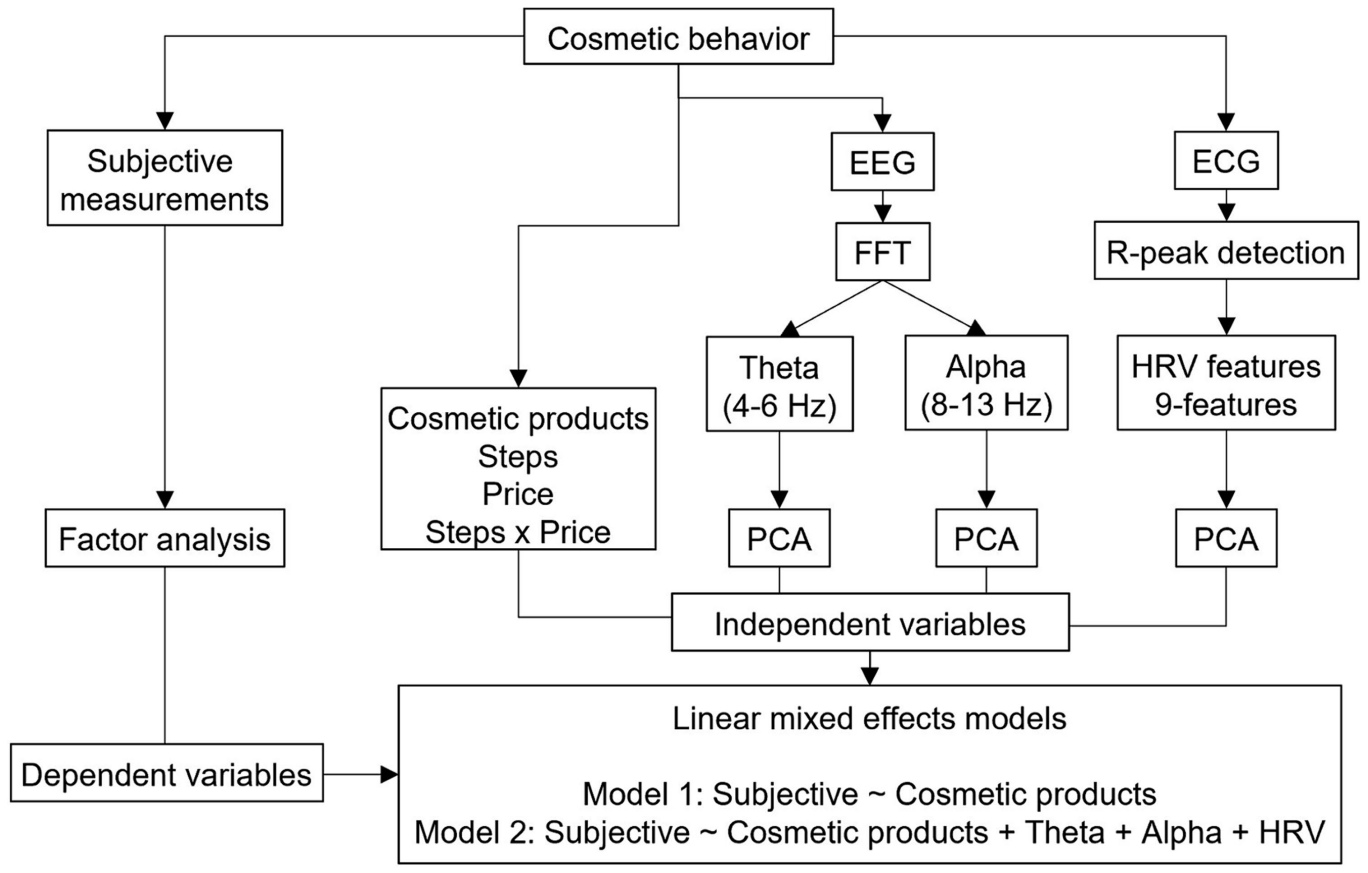
Block diagram of statistical analysis used in the current study.

Statistical analyses were performed using R version 4.1.1 (R Core Team, 2021). Multivariate decomposition analyses and statistical model (linear mixed effects model) fitting were performed using psych 2.1.9 (Revelle, 2021) and lme4 1.1–27.1 (Bates et al., 2015), respectively. A block diagram of the statistical analysis used in the current study is shown in Figure 2.

#### 2.6.1 Exploratory factor analysis for subjective measurements

To find a latent variable that explains the variation of the subjective experience, we performed an exploratory factor analysis on the subjective measurements. The subjective scores were subjected to factor analysis with Geomin Q rotation to identify low-dimensional latent variables. The number of factors was determined by a parallel analysis.

#### 2.6.2 Principal component analysis for physiological measurements

The power of EEG signal obtained at each electrode was highly correlated with each other because of the nature of volume conduction of the EEG signals. Also, individual HRV metrics are correlated with each other because each metric measures similar aspects of temporal variation of heart rate by using different formulas. This should be problematic when a linear model is fitted by causing multiple collinearity problems. We applied a principal component analysis (PCA) with oblimin rotation separately for each physiological measurement (i.e., theta band power, alpha band power, and HRV) to avoid this problem. For both the theta and alpha power, power spectrum values calculated at individual electrodes (18 electrodes) were submitted into PCA with oblimin rotation. For the heart rate variability features, 9-HRV features were submitted into PCA with oblimin rotation. For all PCA, the number of components was determined based on the number of components whose eigenvalues exceeded 1.0.

#### 2.6.3 Linear mixed effects models

To examine whether the subjective responses were explained by the cosmetic products and physiological measurements, we fitted a statistical model using linear mixed-effects models with a restricted maximum likelihood estimation. Because the random effects of participants can be considered, the mixed-effects model is one of the best solutions when dependent variables are repeatedly measured for a participant. The dependent variables were the first, second, and third-factor scores of the subjective measurements, and the independent variables were price, step of cosmetic behavior, two-component scores of theta power, two-component scores of alpha-band power, and two-component scores of heart rate variabilities. The random effects of participants on the intercept were also estimated. Categorical variables (the price, step of cosmetic behavior, and participants) were transformed into a one-hot vector that includes-1, 0, and 1 by using the sum contrast option in R (contr. Sum). Two models were fitted for each of the factors of the subjective measurement: one included the price, step of cosmetic behavior, and interaction of the price and step of cosmetic behavior as independent variables, and the other included the price, step of cosmetic behavior, interaction of the price and step of cosmetic behavior, theta component scores, alpha component scores, and heart rate variability component scores as continuous independent variables. These two models were compared using a likelihood ratio test to determine whether the existence of physiological features could improve the models.

## 3 Results

### 3.1 Factor analysis on the subjective measurements

A parallel analysis applied to the raw scores before the main factor analysis indicated that three factors had the best number of features. Table 1 presents the results of the factor analysis. As a result, 62.6% of the variance was explained by three factors (25.0, 24.2, and 13.5% for Factors 1, 2, and 3, respectively). For Factor 1, the factor loadings were higher for questions related to self-confidence (e.g., *confidence*). For Factor 2, the factor loadings were positively higher for questions related to the user experience of cosmetic products, tactile sensation, and positive emotions (e.g., *the product is easy to use*). The factor loadings for Factor 3 were higher for questions related to negative emotions (e.g., *anxi*ety). We named factor 1, factor 2, and factor 3 as self-confidence, hedonic perception, and negative emotion, respectively.

**Table 1:** The result of factor analysis for subjective measurements.

	Factor 1	Factor 2	Factor 3	Communality	Uniqueness	Complexity
Want to see someone	1.032	−0.011	0.095	0.95	0.05	1.02
Confident	0.934	−0.036	−0.036	0.88	0.12	1.01
A good impression of the entire face	0.698	0.293	−0.046	0.80	0.20	1.35
Concentrated	0.309	0.198	0.070	0.15	0.85	1.83
Feel good to touch the skin	−0.074	0.897	0.021	0.74	0.26	1.01
Easy to use	0.001	0.860	0.068	0.68	0.32	1.01
Cheerful	0.445	0.624	−0.029	0.86	0.14	1.81
Calm	0.225	0.557	−0.102	0.57	0.43	1.39
Refreshed	−0.015	0.498	−0.024	0.25	0.75	1.01
Satisfied	0.342	0.367	−0.328	0.73	0.27	2.97
Anxious	0.001	0.033	0.898	0.77	0.23	1.00
Unpleasant	−0.045	−0.073	0.723	0.63	0.37	1.03
Sleepy	0.066	−0.046	0.361	0.12	0.88	1.10
SS loadings	3.246	3.141	1.756			
Proportion of variances	0.250	0.242	0.135			
Cumulative proportion of variances	0.250	0.491	0.626			
Proportion explained	0.399	0.386	0.216			
Cumulative proportion explained	0.399	0.784	1.000			

### 3.2 PCA on the physiological measurements

**Figure 3:**
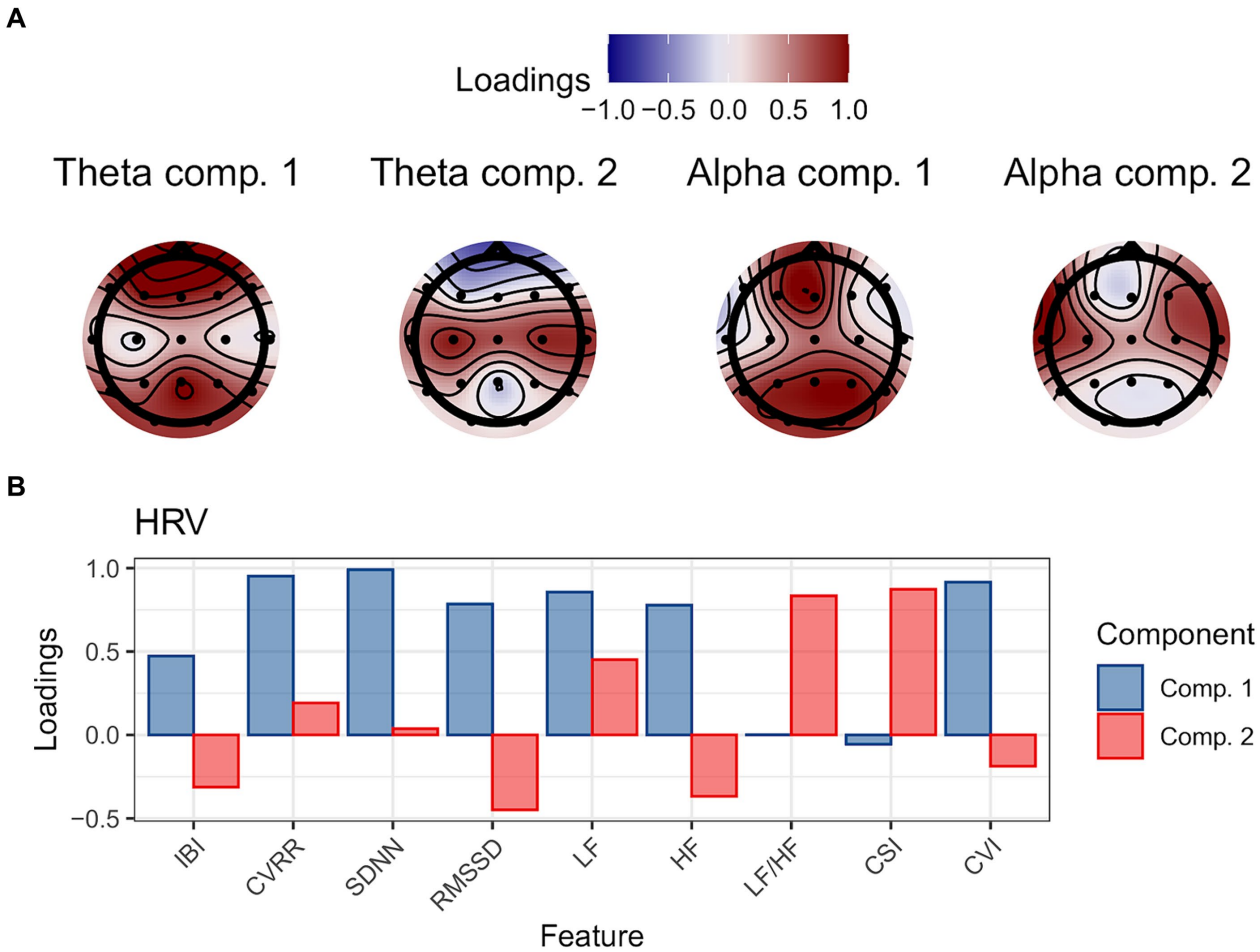
The loadings of PCA for **(A)** theta and alpha-band powers (shown in topographical map), and **(B)** HRV measurements.

The loadings of the PCA results for each physiological feature are shown in Figure 3.

#### 3.2.1 Theta band power

The number of components was 2 based on the criteria of eigenvalue >1.0. For the theta band power, 70.3% of the total variance was explained by two principal components. The rotated component loading of the theta band power was as follows: The rotated component loadings of component 1 were positively greater over the frontal and parietal scalp electrode sites (Figure 3A, far left), and the rotated loadings of component 2 were positively greater over lateral central electrode sites (Figure 3A, second from left).

#### 3.2.2 Alpha band power

The number of components was 2 based on the criteria of eigenvalue >1.0. For the alpha band power, 77.4% of the total variance was explained by two principal components. The rotated component loading of the alpha band power was as follows: The rotated component loadings of component 1 were positively greater over frontal and parieto-occipital scalp electrode sites (Figure 3A, second from right), and the rotated loadings of component 2 were positively greater over lateral frontocentral electrode sites (Figure 3A, far right).

#### 3.2.3 HRV

The number of components was 2 based on the criteria of eigenvalue >1.0. For the alpha band power, 77.4% of the total variance was explained by two principal components. For the heart rate variability, the loadings of component 1 were positively greater for IBI, CVRR, SDNN, RMSSD, LF, HF, and CVI, whereas the loadings of component 2 were positively greater for LF, LF/HF, and CSI and negatively greater for IBI, RMSSD, and HF (Figure 3B).

## 4 Statistical model

### 4.1 Model fitting and model comparison

We fit two linear mixed effect models separately for each factor score of subjective measurements. One model includes only cosmetic steps and price condition and their interaction as independent variables, and the other includes cosmetic steps, price condition, the interaction term of cosmetic steps and price condition, theta band power, alpha band power, and HRV as independent variables. More specifically, for example, the linear mixed models for the self-confidence factor (Factor 1) were as follows:


$$\mathrm{Self}-\mathrm{confidence}\sim \mathrm{Step}+\mathrm{Price}+\mathrm{Step}:\mathrm{Price}+\left(1|\mathrm{Participant}\right)$$

and


$$\begin{array}{l} \mathrm{Self}-\mathrm{confidence}\sim \mathrm{Step}+\mathrm{Price}+\mathrm{Step}:\mathrm{Price}+\mathrm{Thet}a_{PC1}+\\ \mathrm{Thet}a_{PC2}+\mathrm{Alph}a_{PC1}+\mathrm{Alph}a_{PC1}+\\ HRV_{PC1}+HRV_{PC1}\left(1|\mathrm{Participant}\right)\text{.} \end{array}$$

Summaries of the statistical models fitted to explain self-confidence (Factor 1), hedonic perception (Factor 2), and negative emotion (Factor 3) are presented in Table 2. For the models of self-confidence and negative emotion, the likelihood ratio test was not significant, indicating that the model including both the cosmetic categories and physiological measures did not improve the model fitting performance compared to the model including only the cosmetic categories (self-confidence: = 6.51, df = 6, *p* = 0.3676; negative emotion: χ^2^ = 12.48, df = 6, *p* = 0.0520). On the other hand, for the model of hedonic perception, a likelihood ratio test showed that the fitting χ^2^ performance was significantly better in the model that included both the cosmetic categories and the physiological measurements than in the model that only included the cosmetic categories (χ^2^ = 16.14, df = 6, *p* = 0.0130). Hereafter, the models selected using the likelihood ratio test are discussed.

**Table 2:** The results of statistical modeling (only selected models are shown).

	Factor 1		Factor 2		Factor 3	
*Predictors*	*Estimates*	*p*	*Estimates*	*p*	*Estimates*	*p*
Intercept	0.00	1.0000	0.00	1.0000	0.00	1.0000
	(−0.168–0.168)		(−0.210–0.210)		(−0.216–0.216)	
Baseline	−1.05	**< 0.0001**	−0.74	**< 0.0001**	0.766	**< 0.0001**
	(−1.187 – −0.912)		(−0.886 – −0.595)		(0.624–0.909)	
Skincare	−0.334	**< 0.0001**	0.451	**< 0.0001**	−0.068	0.3449
	(−0.471 – −0.196)		(0.310–0.593)		(−0.211–0.074)	
Base makeup	0.046	0.5107	0.184	**0.0114**	−0.037	0.6042
	(−0.091–0.183)		(0.042–0.327)		(−0.180–0.105)	
Eye makeup	0.518	**< 0.0001**	−0.157	**0.0331**	−0.198	**0.0065**
	(0.381–0.656)		(−0.300 – −0.013)		(−0.340 – −0.056)	
Affordable	−0.068	0.0535	−0.165	**0.0000**	0.015	0.6690
	(−0.136–0.001)		(−0.239 – −0.092)		(−0.056–0.087)	
Baseline: Affordable	0.098	0.1632	0.258	**0.0004**	−0.142	0.0504
	(−0.040–0.235)		(0.117–0.400)		(−0.284–0.000)	
Skincare: Affordable	−0.017	0.8035	−0.095	0.1857	0.03	0.6802
	(−0.155–0.120)		(−0.237–0.046)		(−0.112–0.172)	
Base makeup: Affordable	−0.007	0.9211	0.039	0.5874	−0.005	0.9454
	(−0.144–0.130)		(−0.102–0.180)		(−0.147–0.137)	
Eye makeup: Affordable	−0.039	0.5721	−0.119	0.0999	0.042	0.5597
	(−0.177–0.098)		(−0.262–0.023)		(−0.100–0.184)	
Theta Comp. 1			−0.127	0.3713		
			(−0.405–0.152)			
Theta Comp. 2			0.168	0.2489		
			(−0.118–0.453)			
Alpha Comp. 1			0.432	**0.0146**		
			(0.086–0.778)			
Alpha Comp. 2			−0.52	**0.0024**		
			(−0.854 – −0.186)			
HRV Comp. 1			0.048	0.5651		
			(−0.116–0.211)			
HRV Comp. 2			0.135	**0.0451**		
			(0.003–0.268)			
Marginal R^2^ / Conditional R^2^	0.446 / 0.630		0.316 / 0.618		0.198 / 0.560	

The *p-*values below 0.05 are shown in bold letters. For each estimate, 95% confidence intervals are shown in the parentheses.

### 4.2 The effects of cosmetic categories

**Figure 4:**
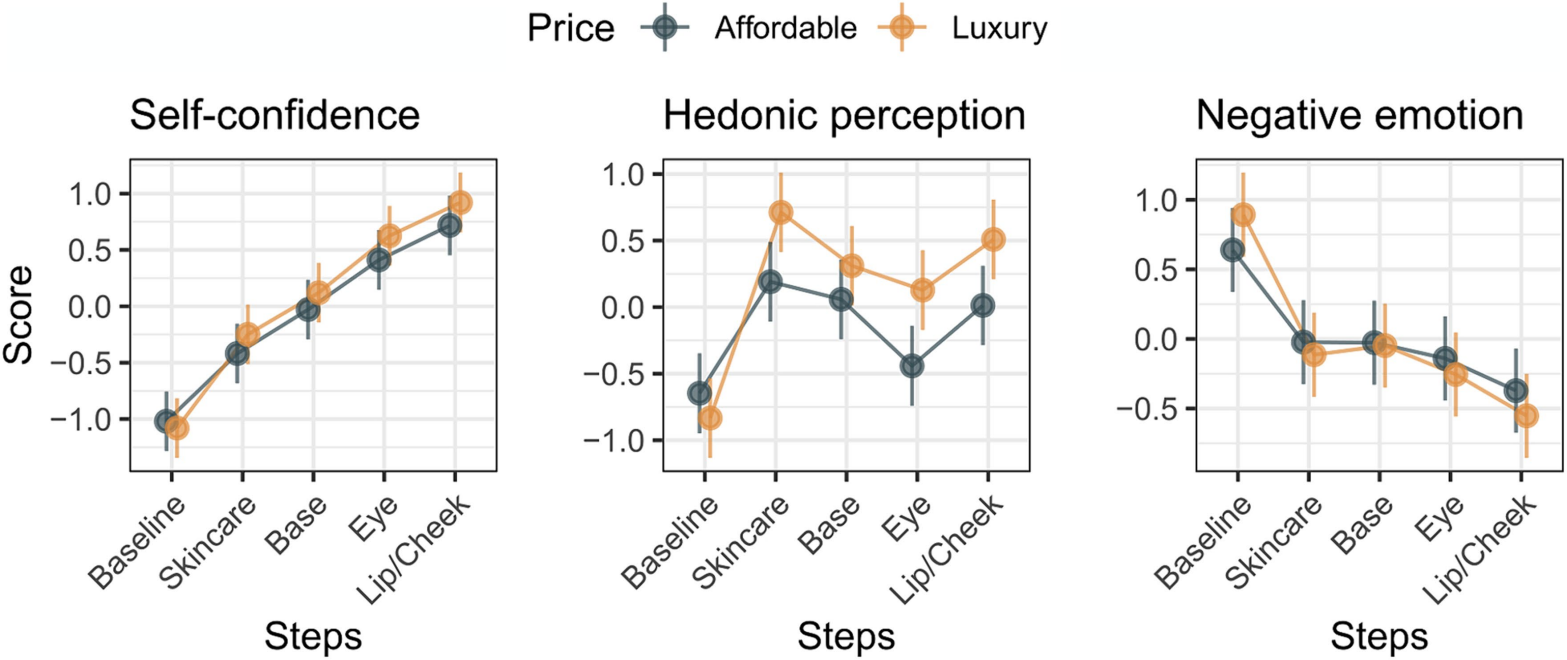
The marginal effects of cosmetic steps and price conditions for each factor score of subjective measurement.

To examine the effects of the cosmetic categories (i.e., price and cosmetic steps), models selected by the likelihood ratio tests were subjected to an analysis of variance (ANOVA) with the Kenward-Roger approximation (Kenward and Roger, 1997). The marginal effects of the categorical variables on the factor scores are shown in Figure 4. For the self-confidence model, the main effect of steps was significant (*F* (4, 261) = 88.56, *p* < 0.0001). The main effect of price and its interaction was not significant for self-confidence (*F*s < 3.76, ps > 0.0535). Multiple comparison tests with Bonferroni correction indicated that the self-confidence score significantly differed among the cosmetic step pairs (ps < 0.0068) except for eye makeup and lip/cheek makeup (*p* = 0.0683). For the hedonic perception model, the main effects of cosmetic steps and price, as well as an interaction effect of the price and cosmetic steps were revealed (main effect of the cosmetic steps: *F* (4, 262.70) = 33.16, *p* < 0.0001; price: *F*(1, 278.02) = 19.47, *p* < 0.0001; interaction effect of price and cosmetic steps: *F* (4, 258.76) = 3.79, *p* = 0.0051). Multiple comparison tests with Bonferroni correction indicated that the hedonic perception scores increased after baseline (ps < 0.0001), then decreased temporarily for eye makeup relative to skincare and base makeup (ps < 0.0350) and increased again for lip/cheek makeup (vs. eye makeup, *p* = 0.0030). The scores for hedonic perception were greater for high-priced products than for low-priced products; however, this effect was significant only for skincare, eye makeup, and lip/cheek steps (*p*s < 0.0024). For negative emotion, the main effect of the number of steps was significant (*F* (4, 261) = 32.43, *p* < 0.0001). The main effects of price and its interaction were not significant (*F*s < 1.10, ps > 0.36). Multiple comparison tests with Bonferroni correction indicated that the negative emotion scores decreased after the baseline (ps < 0.0001) and then further decreased at the lip/cheek makeup step as compared to the skincare and base makeup steps (ps < 0.0066).

### 4.3 Effects of physiological measures

**Figure 5:**
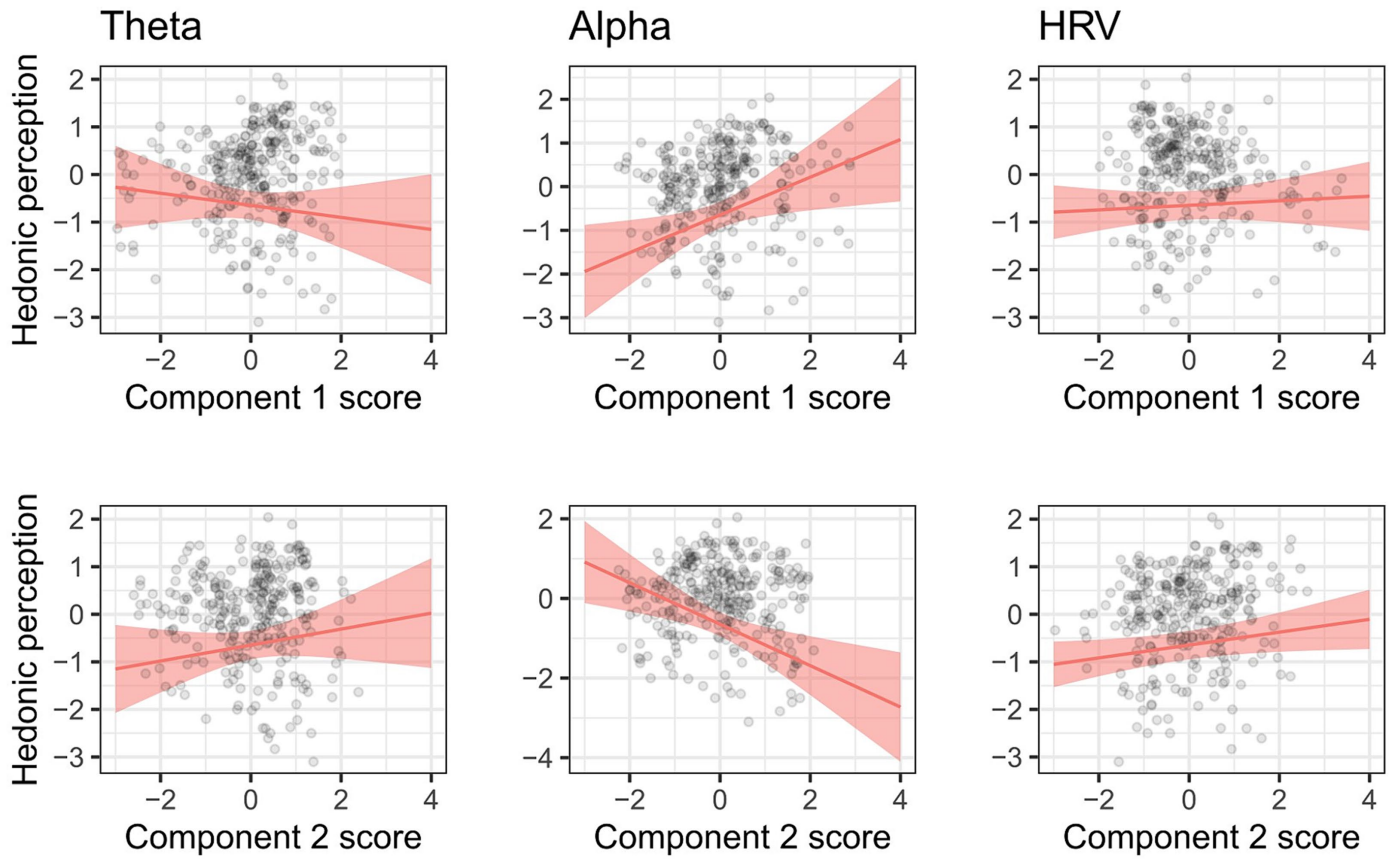
Marginal effects of each component score of theta-band (left), alpha-band (middle), and HRV (right) on the Factor 2 score of subjective measurements. Components 1 and 2 of the physiological measures are depicted in the upper and lower panel, respectively.

The model including physiological measures was chosen only when the hedonic perception score was the dependent variable. The ANOVA showed that the effects of component 1 of alpha-band power (*F* (1, 51.39) = 5.83, *p* = 0.0194), component 2 of alpha-band power (*F* (1, 72.35) = 9.02, *p* = 0.0037), and component 2 of heart rate variabilities (*F* (1, 220.80) = 3.94, *p* = 0.0483) were also significant for the hedonic perception model. The predicted value of the hedonic perception score for each physiological measure is shown in Figure 5. The hedonic perception scores increased as component 1 of alpha-band power and component 2 of the heart rate variability increased. In contrast, the hedonic perception scores decreased as component 2 of the alpha band power increased.

## 5 Discussion

In this study, we examined a variety of cosmetic products and the physiological response that can explain the change in the subjective response during the evaluation of a series of skincare and makeup steps. The subjective measurements were decomposed into three factors using factor analysis. Factors 1, 2, and 3 are likely to reflect self-confidence, hedonic perception, and negative emotions in response to cosmetic steps, respectively. Physiological responses, theta power, alpha power, and heart rate variability were recorded from participants who were asked to look in a mirror after applying each cosmetic item, which was decomposed into two components using principal component analysis. Both theta and alpha band powers were decomposed into components that showed higher component loadings over the frontal, parietal, and occipital electrode sites and components that showed higher component loadings over the temporal and central electrode sites. Heart rate variabilities were decomposed into components that were likely to reflect parasympathetic nervous system activity and components that were likely to reflect sympathetic nervous system activity. The statistical models showed that the features of the cosmetic products influenced the three subjective responses to the cosmetic series; however, the physiological responses influenced only the hedonic perception.

### 5.1 The effects of cosmetic products on the subjective experience

The Factor 1 score, namely self-confidence, linearly increased as the cosmetic series progressed, without any influence of the price of the cosmetic products. This result suggests that the recognition of the modification in facial appearance, including skin, by visual information obtained through the process of skincare and makeup or the makeup completion is important to increase self-confidence felt during or after cosmetic behavior. The Factor 2 score, namely hedonic perception including positive emotions and motor/tactile perception, increased immediately after skincare, decreased after eye makeup, and increased again after lip/cheek makeup. The decrement in hedonic perception in the eye makeup step may be explained by the fact that the tactile innervation density is relatively low around the eyes compared to other parts of the face (Corniani and Saal, 2020). Additionally, in the use of eye makeup, attention was allocated to the application, and the function and finish were emphasized after coating. Therefore, the effects of conscious feelings of texture and associated emotions are weaker in eye makeup than in other cosmetic steps. Importantly, the hedonic perception score was higher for luxury cosmetic products than for affordable cosmetic products, except for the baseline and base makeup steps. The factor loadings of the hedonic perception factor indicated that the score reflected the participants’ evaluation of the tactile sensation of the cosmetic products and the associated positive emotions. These results strongly suggest that participants might be sensitive to differences in texture between affordable and luxury brand products. Differences in EEG and cerebral blood flow responses have been reported for skincare, foundation, and lipstick use as described above (Tanida et al., 2017; Kawabata Duncan et al., 2019; Gabriel et al., 2021; Hirabayashi et al., 2021). The effect of price was not present in the base makeup step, explanation of the reason is quite difficult, because the difference of the price between luxury and affordable price levels was the largest in the base makeup step (see Supplementary Table S1), therefore the difference of price cannot explain this result. One possible reason of the lack of the effect of price is that the brand-specific difference of makeup base and the foundation included in the base makeup step are less likely to be related to hedonic perception that was measured in the current study. For example, one of the functional importance of the foundation is changing color of facial surface, which is not directly measured by questionnaire items (i.e., feel good to touch the skin, the product is easy to use, cheerful, calm, refreshed, and satisfied) included in the hedonic perception, thus hedonic perception factor score might not detect the effect of the price level in the base makeup products. Factor 3, namely, the negative emotion score, decreased immediately after baseline and further decreased as the cosmetic steps progressed. Given that the Factor 3 score reflects negative emotions, including anxiety and sleepiness, this result suggests that participants felt bored or dull at baseline; however, these negative emotions were eliminated by applying the cosmetic items.

In summary, the results of the current study indicate that (1) self-confidence increases as the number of cosmetics used increases, (2) the hedonic perception increases during skincare, base makeup, and lip/cheek makeup, especially when one uses luxury cosmetic products, and (3) negative emotions are eliminated by cosmetic behavior. These findings are in line with previous findings in which it was revealed that one of the reasons for consumers’ use of cosmetic products is to improve self-confidence (Korichi et al., 2008). To the best of our knowledge, this is the first direct evidence that the prices and steps of cosmetic products interactively affect participants’ perceptions and emotions during cosmetic behavior.

### 5.2 The relationships between physiological activities and subjective experiences

The effects of physiological activities were found only in hedonic perception. The model indicates that both the alpha-band components and the second component of heart rate variability can explain the variation in the hedonic perception score.

For alpha band activities, the two components showed opposite effects on the hedonic perception score; for instance, the effect of Component 1 was positive, whereas the effect of Component 2 was negative. From the scalp distribution of component loadings, Components 1 and 2 may reflect the oscillatory activity of the parieto-occipital alpha and mu rhythms, respectively. It is well-known that the alpha-band activities over the occipital region decrease after visual stimulus onset relative to the pre-stimulus period especially when participants’ attention is allocated to the given visual stimulus (Fries et al., 2008; Bollimunta et al., 2011). The mu rhythm is defined by a similar frequency range as the alpha band (8–15 Hz); however, it is predominantly distributed over the lateral central-parietal scalp sites and is thought to reflect the activities of the motor cortices and/or somatosensory cortices (McFarland et al., 2000). Previous studies have shown that the mu rhythm activities decreased during motor execution and motor imagery (McFarland et al., 2000; Pfurtscheller et al., 2006; Frenkel-Toledo et al., 2013), as well as tactile stimulation (Cheyne et al., 2003; Singh et al., 2014), which is relative to the resting or the pre-stimulus period. These findings suggest that the positive emotional/perceptual response after cosmetic behavior is related to increased activity in the sensorimotor cortices and decreased activities in the visual cortices. In other words, the results suggest that the evaluation of the goodness of cosmetic products and consumers’ emotions may be more positive when a participant allocates greater attentional resources to tactile rather than visual information.

For the heart rate variabilities, the second component is positively correlated with the hedonic perception score. Component loading was greater in features such as LF/HF, which are related to sympathetic nervous system activity. The relationship between positive emotions and heart rate variability is slightly controversial. A common understanding is that sympathetic nervous system activity decreases, whereas parasympathetic nervous system activity increases in response to positive emotion elicitation; however, previous findings are less consistent with heart rate variability measures (see Kreibig, 2010). An increased sympathetic activity indexed by heart rate variability is reported (Kop et al., 2011). Kop et al. found that the LF and LF/HF ratios increased while remembering happy memories compared with baseline. Their findings are in line with our results because the analysis time window of the physiological measures in the current study was limited to the periods when the participants looked in the mirror and thought about the completeness of makeup, how the cosmetic products were, and so on, after a series of cosmetic behaviors. However, in daily life, people sometimes identify themselves by looking at their facial makeup, which results from a series of cosmetic behaviors, immediately or hours after applying cosmetics. The results of this study suggest that seeing one’s own face with makeup evokes the experience of cosmetic behavior as well as the perceptual experience of cosmetic integrity, and that positive emotions and sympathetic nervous system activity are induced, which could mean that the gains from cosmetic behavior are sustained not only during the time of the behavior but also afterward. This kind of aftereffect found in the current study also suggests that cosmetic behavior may contribute to people’s well-being involving hedonic emotions. The current results may expand the effect of cosmetic behavior on the emotions and the autonomic nervous systems during cosmetic application by others or self and afterward at rest found in the previous studies (e.g., Tanida et al., 2017; Bouhout et al., 2023).

In summary, the current results indicate that activities in the central and autonomic nervous systems play a role in user experience during cosmetic behavior, especially in the evaluation of the tactile sensation of cosmetic products and the associated positive emotions. The current results did not show any influence of physiological activities on self-confidence or negative emotions as reflected by Factors 1 and 3 of the subjective measures, respectively. One possible reason is that changes in self-confidence and negative emotions are not strong enough to detect the effects of physiological responses. This is plausible for negative emotions because Factor 3 is a combination of anxiety, unpleasantness, and sleep, suggesting that participants felt bored or dull in the baseline period. Another possible reason is that the central nervous system activities measured by EEG did not reflect the participants’ self-confidence and negative emotions. A previous study showed that the cortical source activity of the alpha band is related to trait optimism (De Pascalis et al., 2013), however, to the best of our knowledge, no direct evidence shows that scalp EEG oscillatory activity is related to a transient change in self-confidence. Further studies are required to confirm whether these results can be replicated.

### 5.3 Limitations and future research

We found that subjective measurements increased as a function of progress in cosmetics. However, we could not dissociate the effect of elapsed time from the start of each session from the effect of cosmetic steps because the order of cosmetic steps was fixed throughout the experiment. Thus, one might think that the effect of cosmetic steps on subjective measurements is confounded by time. This is an example of the difficulties in examining the effect of cosmetics on psychological factors because daily cosmetic behavior from a face with no makeup to a face with full makeup progresses from skincare, base makeup, and then makeup for facial parts such as eyes, cheeks, and lips. Further research is required to address this confounding issue. For example, in daily life, people usually reapply makeup to their faces during the day as a makeover, and at night, they perform skincare from make-up remover and face wash to lotion to emulsion. It is also possible to examine the condition of the cosmetic behavior for skin care in such situations.

In the current study, the subjective experiences were compared between luxury and affordable price level to examine the effect of objective value of the cosmetic products, however, evidence from behavioral economics studies emphasized that subjective value plays an important role in the economic decision making (e.g., Aljazzazen and Balawi, 2022). Although the result of current study suggests that price of the cosmetic products may one of the factors that influence the subjective value, we did not measure a direct measure of the subjective values such as preference and willingness to pay of the given products, thus (1) whether subjective values varies among each cosmetic step and price level, and (2) whether the other factors except for price, such as users’ prior knowledge, uses’ expectations, and physical characteristics of cosmetic products can influence the subjective values, remain unclear. Further research in which examine detailed influences of various aspects of cosmetic products on the uses’ subjective values, and develop and test a computational model that may help cosmetic manufacturers’ products development are needed.

Another limitation is that the physiological measures used in the current analysis did not directly reflect the participants’ emotional or perceptual experiences during the series of cosmetic behaviors. This is due to the weakness of EEG measures of bodily/electrode motions. In the current experiment, we decided to analyze physiological measures within the time windows where participants watched their faces immediately after the given cosmetic steps to avoid artifacts that are expected to occur during cosmetic behavior as much as possible; therefore, the current results must be interpreted as an aftereffect rather than a direct effect of cosmetic behavior on consumers’ perceptions and emotions. Recent advances in EEG data acquisition systems and analysis using a machine-learning approach (i.e., mobile body/brain imaging: MoBI) have enabled researchers to analyze and interpret EEG signals recorded when participants actively move in more naturalistic circumstances, such as walking (Richardson et al., 2022). Using high-density EEG recordings integrated with head-and-hand motion sensors may help overcome the lack of ecological validity in the present study.

Nevertheless, the current results indicate that some aspects of customers’ subjective emotional and/or perceptual experiences, which are elicited by a series of cosmetic behaviors, can be explained by physiological activities, including alpha-band EEG activities and HRV. This finding suggests a prediction model in which emotional responses can be created as a biomarker of the user experience during cosmetic behavior. Such biomarkers may help to develop new cosmetic products and plan marketing strategies. This possibility can be tested by correcting datasets using a larger sample size.

## 6 Conclusion

The current results show that part of the subjective user experience during cosmetic behavior can be explained by both cosmetic items and physiological responses. Self-confidence increased and negative emotions decreased as the cosmetic steps progressed; however, these were not affected by the price of cosmetic items or physiological activities. On the other hand, the subjective evaluation of tactile perception and the positive emotions induced by applying cosmetic products were influenced by both the variation of the cosmetic items (i.e., steps and price) and physiological responses. Luxury cosmetic products have been evaluated as positively comparable to affordable products; however, this effect is not observed for eye makeup. The positive tactile and emotional evaluations were explained using physiological measures. Alpha-band activity over the lateral central electrode sites was negative, alpha-band activity over the occipital electrode sites was positive, and sympathetic activity indicated by the HRV was positively related to the positive evaluation of tactile perception and emotion. These results indicate that tactile sensory processing in the sensorimotor cortical area of the brain and sympathetic autonomic nervous system activities play important roles in users’ positive experiences during cosmetic behavior. Furthermore, the findings of the current study suggest that physiological measurements can be used as objective indices of the effectiveness of cosmetic products.

## Data availability statement

The datasets presented in this article are not readily available because of confidentiality agreements with the participants. The data in this study are available only at Centan Inc. and Shiseido Co., Ltd. Requests to access the datasets should be directed to KT, keiko.tagai@shiseido.com.

## Ethics statement

The studies involving humans were approved by Ethics committee of the MIRAI Technology Institute, Shiseido Co., Ltd (the human study number is C01967). The studies were conducted in accordance with the local legislation and institutional requirements. The participants provided their written informed consent to participate in this study.

## Author contributions

HM, AM, TM, TH, and KT planned the experimental design, prepared cosmetic materials, and managed the experiments. HM and TH prepared and conducted the experiments, analyzed the data. HM and KT wrote the manuscript. KT revised the manuscript. All authors contributed to the article and approved the submitted version.
