# Supplementary material for: Relationships between subjective experience, electroencephalogram, and heart rate variability during a series of cosmetic behavior
Source: Front Psychol. 2024 May 14;15:1225737. doi: 10.3389/fpsyg.2024.1225737 (PMC11130498; doi:10.3389/fpsyg.2024.1225737)
Supplement: Supplementary file 1 [file Table_1.docx]

Supplementary Material

# Supplementary Figures and Tables

Table S1. List of the cosmetic items used in the current study.

| Cosmetic step condition | Price condition | Brand | Function/Color | Type | Price (Japanese yen) for experimental use |
| --- | --- | --- | --- | --- | --- |
| Skin care | Affordable | Aqua label | Whitening | Lotion | ¥1,400 |
| Skin care | Affordable | Aqua label | Moisturizer | Lotion | ¥1,400 |
| Skin care | Affordable | Aqua label | Whitening | Emulsion | ¥1,500 |
| Skin care | Affordable | Aqua label | Moisturizer | Emulsion | ¥1,500 |
| Base makeup | Affordable | Integrate | Makeup base | Liquid | ¥1,000 |
| Base makeup | Affordable | Integrate | Light ochre | Liquid | ¥1,300 |
| Base makeup | Affordable | Integrate | Natural ochre | Liquid | ¥1,300 |
| Base makeup | Affordable | Integrate | Not available | Powder | ¥1,300 |
| Eye makeup | Affordable | Integrate | Brown | Eye blow | ¥700 |
| Eye makeup | Affordable | Integrate | Gray | Eye blow | ¥700 |
| Eye makeup | Affordable | Integrate | Beige/brown | Eye shadow | ¥850 |
| Eye makeup | Affordable | Integrate | Pink/Burgundy | Eye shadow | ¥850 |
| Eye makeup | Affordable | Integrate | Brown | Gel eye liner | ¥950 |
| Eye makeup | Affordable | Integrate | Black | Gel eye liner | ¥950 |
| Eye makeup | Affordable | Integrate | Volume | Mascara | ¥1,200 |
| Eye makeup | Affordable | Integrate | Long | Mascara | ¥1,200 |
| Lip and cheek makeup | Affordable | Integrate | Natural red | Lipstick | ¥1,200 |
| Lip and cheek makeup | Affordable | Integrate | Natural pink | Lipstick | ¥1,200 |
| Lip and cheek makeup | Affordable | Integrate | Pink | Cheek | ¥1,500 |
| Lip and cheek makeup | Affordable | Integrate | Orange red | Cheek | ¥1,500 |
| Skin care | Luxury | Clé de peau beuté | Moisturizer | Lotion | ¥10,500 |
| Skin care | Luxury | Clé de peau beuté | Clear | Lotion | ¥10,000 |
| Skin care | Luxury | Clé de peau beuté | Daytime | Emulsion | ¥11,000 |
| Skin care | Luxury | Clé de peau beuté | Nighttime | Emulsion | ¥13,000 |
| Base makeup | Luxury | Clé de peau beuté | Makeup base | Liquid | ¥6,000 |
| Base makeup | Luxury | Clé de peau beuté | Light ochre | Liquid | ¥30,000 |
| Base makeup | Luxury | Clé de peau beuté | Natural ochre | Liquid | ¥30,000 |
| Base makeup | Luxury | Clé de peau beuté | Not available | Powder | ¥12,000 |
| Eye makeup | Luxury | Clé de peau beuté | Brown | Pencil eye blow | ¥2,000 |
| Eye makeup | Luxury | Clé de peau beuté | Gray | Pencil eye blow | ¥2,000 |
| Eye makeup | Luxury | Clé de peau beuté | Pink brown | Eye shadow | ¥5,000 |
| Eye makeup | Luxury | Clé de peau beuté | Yellow brown | Eye shadow | ¥5,000 |
| Eye makeup | Luxury | Clé de peau beuté | Black | Liquid eye liner | ¥5,000 |
| Eye makeup | Luxury | Clé de peau beuté | Brown | Liquid eye liner | ¥5,000 |
| Eye makeup | Luxury | Clé de peau beuté | Black | Mascara | ¥5,000 |
| Eye makeup | Luxury | Clé de peau beuté | Brown | Mascara | ¥5,000 |
| Lip and cheek makeup | Luxury | Clé de peau beuté | Camellia | Lipstick | ¥6,000 |
| Lip and cheek makeup | Luxury | Clé de peau beuté | Rose | Lipstick | ¥6,000 |
| Lip and cheek makeup | Luxury | Clé de peau beuté | Pink rose | Cheek | ¥4,500 |
| Lip and cheek makeup | Luxury | Clé de peau beuté | Orange | Cheek | ¥4,500 |

**
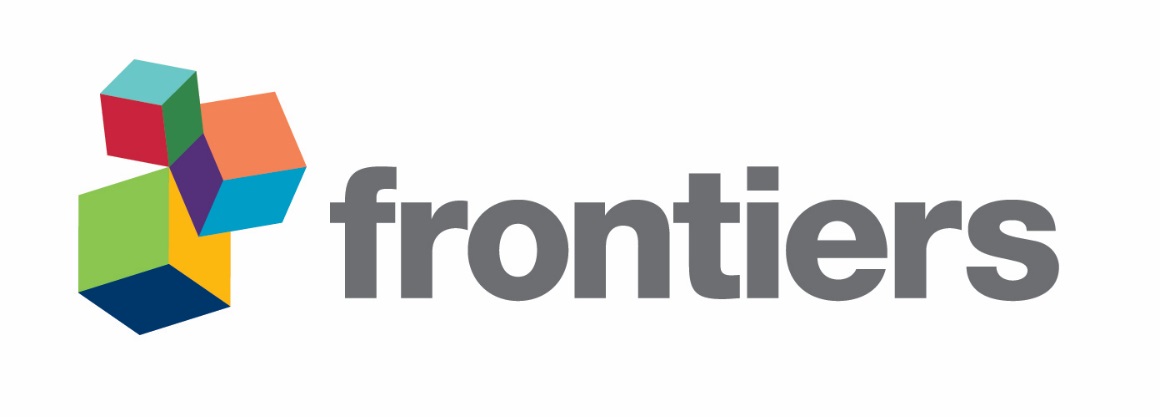
**
